# Systematic Review: Patient and Public Involvement of Children and Young People in Mental Health Research

**DOI:** 10.1007/s10567-024-00470-x

**Published:** 2024-02-25

**Authors:** Christina Totzeck, Anna Swantje van der Meer, Hanna Christiansen, Friederike Durlach, Kira Li Sanchez, Silvia Schneider

**Affiliations:** 1 Mental Health Research and Treatment Center (FBZ), Ruhr University Bochum, Massenbergstrasse 9-13, 44787 Bochum, Germany; 2 Department of Child and Adolescent Psychology, Philipps University Marburg, Marburg, Germany; 3 German Center for Mental Health (DZPG), Partner Site Bochum/Marburg, Bochum, Bochum, Germany

**Keywords:** Patient and public involvement, PPI, Mental health research, Childhood and adolescence, Participation

## Abstract

**Objective:**

Patient and public involvement (PPI) is an essential ethical component in mental health research, and represents a major opportunity to improve translational mental health research. The goals of this review were to (1) provide a comprehensive overview of empirical research focusing on PPI of children and young people (CYP) in mental health research studies; (2) evaluate the results with CYP and parents of those affected; and (3) derive recommendations for PPI of CYP in future mental health research studies.

**Methods:**

Based on an extensive literature review following the PRISMA guidelines, studies including CYP (age range: 0–21 years) in mental health research were identified and examined along a two-part analysis process considering their usability for mental health research. The conclusions drawn from the studies concerning CYP involvement were summarized and recommendations derived.

**Results:**

Overall, 19 articles reported PPI of CYP (age range: 10–26 years) in mental health research and were included for further analyses. The integrated studies differed in the type of PPI, and in the way the participation and involvement processes were presented.

**Conclusion:**

Progress has been made in engaging CYP in mental health research, but there is a need for international standards, operationalization, and evaluation measures. Future research should go beyond merely reporting the PPI process itself. It should clearly indicate how and to what extent feedback from these PPI members was incorporated throughout the research process.

**Supplementary Information:**

The online version contains supplementary material available at 10.1007/s10567-024-00470-x.

## Introduction

Patient and public involvement (PPI) has become a core element in health research (Brett et al., 2014; Brett et al., 2014; McCoy et al., 2019). As defined by the United Kingdom’s (UK) leading participation foundation INVOLVE, PPI describes “research being carried out ‘with’ or ‘by’ members of the public rather than ‘to’, ‘about’ or ‘for’ them” (INVOLVE, 2012). This is not limited to a specific step in the process, but includes the involvement of those affected in every part of the planning, concept development and conduction of research studies. The aim of participatory research is thus to dovetail science and society in order to enable new forms of knowledge (Unger, 2014).

The roots of PPI in mental health research can be traced back to the 1960s and 1970s, when to our knowledge the first advocacy groups were established that sought to promote the rights of patients and their families and to ensure that their voices were heard in decisions about their care (Mold, 2012; Rose, 2022). In response, researchers began to develop methods for involving patients in the research process, including consultation, collaboration, and co-production (sharing power and responsibility from the start of the project). Although often used simultaneously, *patient participation* and *patient involvement* are two distinct concepts that highlight different levels of engagement and collaboration between healthcare providers and patients (Thompson, 2007). Patient participation refers to patients’ active engagement in their own care, such as sharing their symptoms, concerns, and preferences with healthcare professionals. It emphasizes patients’ role as active participants in the decision-making processes, and encourages them to provide feedback and to ask questions. On the other hand, patient involvement goes a step further by including patients in the planning, design, and evaluation of healthcare services and policies. It recognizes the value of incorporating patients' perspectives and experiences into the overall healthcare system, shaping it to be more patient-centered and responsive to individual needs. Patient involvement fosters a sense of ownership and empowerment among patients, promoting a collaborative relationship between patients and healthcare providers for improved health outcomes.

One of the key milestones in the history of patient and public involvement in mental health research was the establishment of the Mental Health Research Network (MHRN) (UK Mental Health Research Network. Guidance for Good Practice: Service User Involvement in the UK Mental Health Research Network, 2005.http://studymore.org.uk/surgea.pdf.Accessed May 27, 2023,2005) in the UK, which was set up to improve the quality and relevance of mental health research by involving patients and the public in the research process. The MHRN brought together mental health researchers and patients to work together on research projects, and provided training and support to researchers on how to involve patients and the public in their work. Another important milestone was the publication of the INVOLVE guidelines (INVOLVE, 2012), which were developed by the UK National Institute for Health Research to provide practical advice on how to involve patients and the public in research. They cover all aspects of the research process, from developing research questions to disseminating research findings.

Although such active involvement of affected patients, their relatives and/or the public has the potential to increase the acceptance and outcome of evidence-based research studies, PPI has seldom been implemented in clinical-psychological, psychotherapeutic and psychiatric research (Peter, 2017). In addition to financial costs and time-consuming effort, the fear of losing control regarding the research process also appears to be a barrier for implementing PPI (Dziobek & Lipinski, 2021). CYP involvement in research is therefore even more sparse. Particularly in childhood and adolescence, there is an urgent need to move away from paternalistic approaches to mental health research towards approaches focusing on CYP and their lived experiences. This research has been neglected in the field of CYP, or limited to questions about research designs and implementation. However, PPI incorporates many more components, such as identifying research needs within the group of people affected, or participating in the development of research questions. Thus, improving PPI in CYP urgently requires model-driven and age-appropriate implementation of a systematic research strategy.

Focusing on mental health research, by far the highest percentage of studies is still carried out in adults, despite the fact that childhood and adolescence are of particular importance: 50% of mental disorders have an onset before the age of 11 years and 75% before the age of 21 years (Kessler et al., 2005). Furthermore, mental disorders of childhood and adolescence/young adulthood are pacemakers for disorders in adulthood (Blakemore, 2019; Kessler et al., 2005). Among children aged 5–14 years, mental disorders have already reached second place among the causes of disability-adjusted life-years (DALYs) (Baranne & Falissard, 2018). The pharmacological (see e.g. Correll et al. 2021) and psychotherapeutic treatment of mental disorders in childhood and adolescence has become more effective in recent decades. In particular, cognitive-behavioral therapy (CBT) shows high efficacy and strong effect sizes in the treatment of several mental disorders, such as pediatric depression (Arnberg & Ost, 2014), anxiety disorders (In-Albon & Schneider, 2007), or post-traumatic stress disorder (PTSD) (Lenz & Hollenbaugh, 2015). Although the strength of effect sizes in the treatment of childhood and youth mental disorders demonstrate high treatment success, there is still room for further improvement (Weisz et al., 2017). Not all patients respond equally well to CBT, and relapse rates in CYP are not yet satisfactory (In-Albon & Schneider, 2007). Further strategies to optimize research and therapy are thus needed. The direct involvement of those affected, in this case CYP, could yield helpful information and thus contribute to better outcomes in both mental health research and the treatment of mental disorders.

Initial research steps in PPI with CYP appear to have been taken in the last two decades (Sellars et al., 2021). A systematic literature search and critical evaluation of previous studies is needed as a starting point in order to initially define and analyze hurdles and obstacles as well as to identify the most effective forms of PPI (Schelven et al., 2020). This applies even more so to PPI of CYP, as this presents us with special challenges: CYP undergo a rapid personal development; they have specific and varying interests and individual, dynamic lives (Handa et al., 2023). They are also confronted with being dependent on their caregivers’ agreement and calendar, as well as balancing their own activities, such as sports, social and peer-group activities, in addition to school and education. All of this results in more fluctuation and less adherence in meetings conducted in the participation process (Mawn et al., 2015). There also appears to be a higher risk of dropping out because of CYP losing interest, fear of stigmatization, and of loss of respect from their peer groups (Schelven et al., 2020).Therefore, mental health research involving CYP needs particular guidance. In addition to basics, such as age-specific language and presentation of information, improving PPI of CYP urgently requires a structured, model-driven and age-appropriate approach. The objective of this current review is therefore, to gain more insights into current PPI processes and barriers and to enhance PPI of CYP in mental health research.

To achieve this, our review has three major goals. First, we present a systematic review of empirical research focusing on the active involvement of CYP in the planning and conduction of mental health research studies. Second, we summarize and analyze the past studies with regard to specific PPI processes and barriers. To deepen our understanding of age-specificity needed to conduct PPI of CYP, we evaluate the results with CYP as well as with parents of CYP who have experienced or are experiencing a mental disorder. Third, we provide recommendations for PPI of CYP in future mental health research studies.

## Method

This systematic review is based on the Preferred Reporting Items for Systematic reviews and Meta-Analyses (PRISMA) Statement (Moher et al., 2009), and the PRISMA-P Statement (Moher et al., 2015). Our review was pre-registered with PROSPERO (International prospective register of systematic reviews, PROSPERO-ID: CRD42022325474).

### Selection of Studies

A literature search was carried out for studies that were published from 2000 (based on work by Sellars et al. (Sellars et al., 2021)) to 2023. We employed the PICO model to define the key elements in our research, which included empirical studies that have engaged CYP (P—Participants) as contributors to mental health research (I—Intervention). We compared the forms of participation and/or involvement (Comparator) and analyzed the information (i.e., experiences, recommendations, barriers) about PPI (Outcomes). The following databases were systematically searched for publications written in English or German: PsycInfo, PSYINDEX, Web of Science and PubMed. Additionally, Google Scholar, and article reference lists of relevant studies were searched as well. Terms within a category were linked with “or” whereas terms between categories were linked with “and”, such as ((Patient participation[Mesh]) OR (community-based participation research[Mesh])) OR ((patient involvement[Title/Abstract]) OR (public involvement[Title/Abstract]) OR (patient engagement[Title/Abstract]) OR (PPI[Title/Abstract]) OR (participatory research[Title/Abstract])). Adaptations to the terms were implemented according to the search style of each database. The search took place in January 2022 and April 2023. Two clinical psychologists on the study team independently screened abstracts. They performed data extraction to gather relevant information (first author; year of publication; country; PPI sample size; age range; sociodemographic background; PPI methodology; intensity of participation; extent of involvement) systematically from each eligible study. No software or automation tools were used in the screening process. Table 1 provides an overview of study details. Overall, four interdisciplinary reviewers (CT, ASvdM, FD, KLS) screened full-texts against the pre-specified inclusion criteria. Disagreements at the study selection process were resolved by discussion among the research team.

**Table 1:** Overview

Study First author, year	Country	PPI sample size *N* (Female)	Age range	Sociodemographic background A) Clinical (disorder)/non-clinical Sample (B) Ethnicity (C) Education	PPI methodology	Intensity of participation Brett et al. (2014) 1–9	Extent of involvement 1–5
Brady, 2018 (Brady et al., 2018)	UK	17 (12)	16–21 years	A) Clinical (alcohol and/or drug misuse) B) Urban and rural background (the West Midlands, the North East and the West of England, London and Oxfordshire) C) No information	YPAG	6	4
Dennehy, 2019 (Dennehy et al., 2019)	Republic of Ireland	16 (10)	16 years	A) Nonclinical B) No information C) Educational diversity	Lundy’s model of participation (2007)	6	4
Dewa, 2021 (Dewa et al., 2021)	UK	7 (5)	18–25 years	A) Clinical (Depression, anxiety disorder, bipolar disorder, anorexia nervosa, psychosis, substance misuse and personality disorder) B) 5 White-British; 1 British-Asian; 1 Black-British C) No information	YPAG and GRIPP2 checklist	7	4
Dunn, 2017 (Dunn, 2017)	UK	17 (12; one gender fluid)	17–22 years	A) Clinical (Depression/anxiety/ self-harm; psychotic illness; autism/anxiety/depression/pain condition; young carer/anger/family; anorexia nervosa/depression/anxiety/auditory/ visual hallucinations; depression/ Asperger/self-harm) B) No information C) 7 students, 4 unemployed	None	7	4
Grant, 2020 (Grant et al., 2020)	UK	26 (no information)	16–18 years	A) Nonclinical (Convenience student sample) B) No individual demographic information to ensure anonymity C) 2 diverse schools	YPAG	6	4
Grové, 2021 (Grové, 2020)	Australia	40 (30; 10 no information)	15–17 years	A-C) No information	None	6	3
Halsall, 2021 (Halsall et al., 2021)	Canada	-Peer-staff: 8 peers, 15 other participants-second round: 7 peers (no information)	14–26 years	A-C) No information	None	5	4
Juan, 2022 (Juan et al., 2021)	UK	Total 68 (45); 46 young people	11–25 years	A) Clinical (current mental health problems 30%, past experiences 30%) and nonclinica^la^ B) Ethnicity: White British/European 44 (65%), Other 20 (30%)^a^ C) No information	YPAG	5	4
Kendal, 2017 (Kendal et al., 2017)	UK	11 (9)	16–18 years	A-C) No information	None	8	4
Lincoln, 2015 (Lincoln et al., 2015)	USA	6 (no information)	18–25 years	A) Clinical (lived experiences of mental health and recovery) B) Ethnicity: self-identified as African-American, Asian and White C) Educational diversity	CBPR	7	5
Mawn, 2015 (Mawn et al., 2015)	UK	Approx. 20 (no information)	14–24 years	A) Clinical and Nonclinical (mental health problems themselves or carer/ sibling of someone with mental health problems or have had no personal experience of mental health issues) B) No information C) No information	Shared decisions approach with youth Hart (1992)	8	5
Miller, 2021 (Miller et al., 2021)	USA	12 (no information)	16–24 years	A) Clinical (Refugee Trauma and Resilience Center) B) Representatives of various ethic and refugee groups C) No information	CBPR	5	3
Ospina-Pinillos, 2018 (Ospina-Pinillos et al., 2018)	Australia	18 (9)	16–25 years	A-C) No further information	PD	8	4
Pfister, 2021 (Pfister et al., 2021)	Switzerland	10 (6)	14–18 years	A) Nonclinical B) With and without history of migration; rural and urban communities in the Canton of Zug C) Youth of all school types in grade/ shortly before finishing their official schooling/ youth in between school graduation and job training/college/ youth in job-training	Stage model of participation Wright et al. (2019)	7	5
Pullmann, 2013 (Pullmann et al., 2013)	USA	3 (no information)	17–19 years	A) Clinical (History of substance use or other behavioral health treatment) B) No information C) No information	PAR and CBPR	6	4
Schilling, 2021 (Schilling et al., 2020)	Chile	6 (3)	14–17 years	A) Clinical (experiences related to adolescent mental health and suicide) B) No information C) High school students	PAR	6	3
Walker, 2021 (Walker et al., 2021)	UK	8 (3 male, 5 no information)	10–17 years	A) Clinical (Neurological or rheumatic diseases, affecting mental health and emotional wellbeing) B) No information C) No information	INVOLVE (2012) CYPAG	6	4
Warner, 2021 (Warner et al., 2021)	Sweden	4 refugee advisors: 3 parents and 1 youth (no information)	No information	A) No information B) Refugee advisors were selected based on how close their personal situation matched that of the intended study participants (i.e. children aged 8 or above showing symptoms of post‐traumatic stress who have resided in Sweden for 5 years or less) C) No information	The Active Involvement of Users in Research Observation Schedule (Schulz et al. 2003)	4	3
Wright, 2019 (Wright et al., 2019)	Australia	No information	No information	A) No information B) Nyoongar Elders and Aboriginal and Torres Strait Islander C) Young people working	PAR	6	4

*YPAG* young people advisory group, *GRIPP2* guidance for reporting involvement of patients and the public, *CBPR* community-based participatory research, *PD* participatory design, *PAR* participatory action research

^a^This information reflects the total sample, no detailed information was provided regarding the young people’s sample

### Criteria for Inclusion

**Fig. 1:**
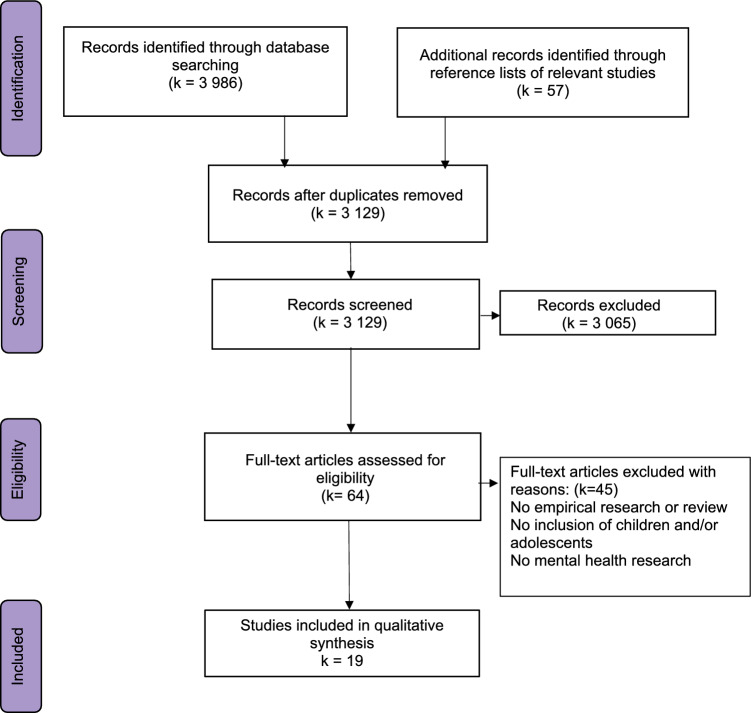
Flow diagram according to PRISMA Guidelines

For this review, we included all original studies in which PPI of CYP took place in a mental health research setting as part of the research team. Only original and published papers were eligible for inclusion. Meta-analyses, scoping or narrative reviews were not included in our review. Papers had to be written in English or German. Main criteria for inclusion were study information reported and responding to these three research questions: (1) “How are children and young people involved in mental health research?”, (2) “How is PPI of children and adolescents reported in mental health research?”, (3) “Which recommendations were generated relying on PPI of children and adolescents in mental health research?”. We set no restrictions regarding sample size or population characteristics (i.e., clinical, non-clinical) of included studies, nor the way PPI was conducted (i.e., co-production, co-design). Age range was defined as CYP from birth to young adulthood (0–21 years), allowing studies of adolescents of a wider age range (i.e., 18–29 years) to also be analyzed. Study designs were not limited; we included quantitative, qualitative and mixed-methods studies (Fig. 1).

### Rating PPI Methodology

**Fig. 2:**
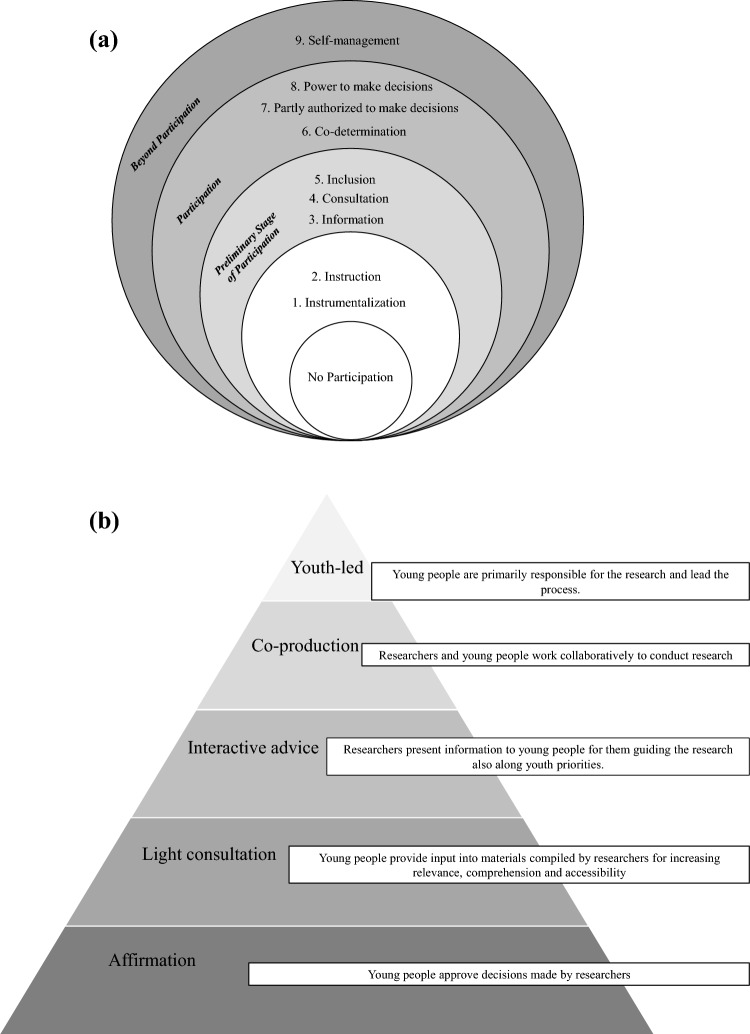
**a** Stage model of participation by Wright (2010) Adapted from Duarte et al. (2018) **b** Levels of involvement, adapted from Sellars et al. (2021)

Due to the heterogeneity of the study designs and data available, we did not perform standardized quality assessments of the research studies. Instead, all included studies were evaluated with regard to their PPI methodology: (1) the participation level as well as (2) the extent of CYP involvement. These ratings are not to be considered as a measurement of the quality of the participation, but are intended to reflect the extent and process of PPI in general. (1) To assess the level of participation in the identified studies, we employed the step-model by Wright et al. (2019) (see Fig. 2a) to arrive at an evaluation of the participation described in a given study. The nine Levels of Participation in PPI as described by Wright et al. (2019) is a framework that outlines various levels of patient and public participation in healthcare research. These levels provide a framework for thinking about the different ways in which patients and the public can participate in healthcare research, and can help to ensure that their contribution is appropriate and meaningful. The aim is to promote greater partnership and collaboration between researchers, patients, and the public, leading to more relevant and impactful research outcomes. While the model was not originally set up with the intention to evaluate the extent of participation, it served as a proxy for study classification in our case.

(2) In addition, the level of involvement was rated based on work by Sellars et al. (2021) (see Fig. 2b) who refer to Arnstein (1969) Hart (1992), and Faithfull et al. (2019). This 5-steps-

**Fig. 3:**
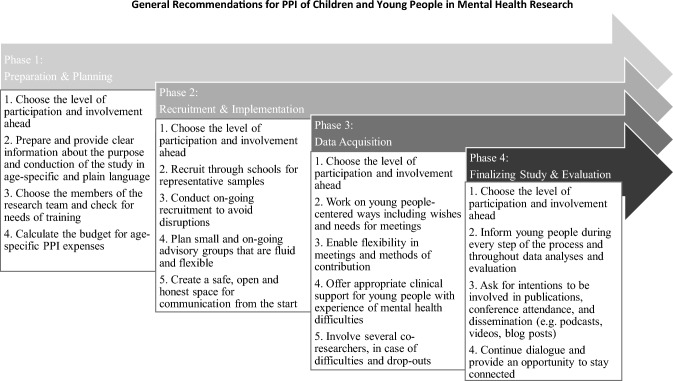
General recommendations for PPI of CYP

The recommendations extracted from the included studies were clustered in four different research steps (Planning, Recruitment, Data Acquisition and Evaluation) and translated into German, respectively plain and age-specific language to enable discussions in our three Advisory Boards: (a) Patients and Relatives (*n* = 7), here specifically parents whose children experience or have experienced a mental disorder provided their feedback in 3 one-hour online meetings, (b) our Children’s Council Mental Health involving children aged 6 to 11 years (*n* = 5) discussed the results in one meeting lasting 90 min, and (c) our Youth’s Council (*n* = 3), involving adolescents aged 12 to 19 years gave their feedback in a 90-min group discussion. All three PPI groups were asked to evaluate the recommendations drawn from the included studies (e.g..: “Do you think that is easy to implement, or are there any difficulties?”; “In conclusion, would you say this is a sensible recommendation?”). These recommendations were presented via Power Point-presentation and discussed separately in each Advisory Board. The participants provided their feedback and added input. These specific recommendations are added and marked in the results section (see Table 2). Finally, in collaboration with the three Advisory Boards, we summarized and prioritized the most important recommendations for future PPI research with CYP (see Fig. 3).

**Table 2:** Recommendations

Study phase	Recommendations
Phase 1	**Preparation:** Choose an appropriate level of participation before the beginning of the study Dennehy et al. (2019), Brady et al. (2018) Plan the study design as demand-driven as possible Mawn et al. (2015), Schilling et al. (2020), Lincoln et al. (2015) Share clearly stated aims with all members including role assignment and task explanation Dunn (2017), Dewa et al. (2021) Plan primary outcomes: Which aspects will be addressed by PPI members? How will their input be recorded? (Review based) *Working with a PPI guideline, as well as train the research team on how to interact with youth (Youth council)* **Planning:** Provide clear and accessible information on the purpose and implementation of the study Walker et al. (2021) Explain how PPI is integrated into the research design Walker et al. (2021) **Prepare study information and informed consent for parents in plain language, respectively native language when recruiting specific ethnicities or otherwise needed (Parents Advisory Board)** Enable skill based mentoring, training or supervision for all members of the team Mawn et al. (2015), Warner et al. (2021) Consider the involvement of a PPI facilitator to support moderation, methods anddissemination Pfister et al. (2021), Walker et al. (2021) Plan sufficient time buffers for possible delays Pfister et al. (2021) Motivate each other if there are barriers at first glance Warner et al. (2021) Utilize youth friendly locations Mawn et al. (2015) Organize refreshments to aid concentration and to provide a comfortable environment Mawn et al. (2015), Dewa et al. (2021) Use agendas for each meeting, so everyone has a timeline; for young children with pictures instead of phrases (Children’s Council) **Budget:** Calculate an adequate budget (considering travel cost, mentoring, training sessions), e.g., using the INVOLVE calculator Mawn et al. (2015), Pfister et al. (2021), Dewa et al. (2021) Include team building activities in the research budget Mawn et al. (2015), Pfister et al. (2021)
Phase 2	R **ecruitment:** Consider age-, gender, and cultural specificities when recruiting Grant et al. (2020) Recruiting in schools, as early as possible in research process is beneficial and is more likely to result in a representative sample Dennehy et al. (2019) Put up posters at school so that children and adolescents can inform themselves and make voluntary decisions to participate (Children’s Council) *Recruitment should also be carried out in youth groups, social media and youth clubs (Youth council)* On-going recruitment is necessary, to minimize disruption caused by the complex and dynamic lives of young people Mawn et al. (2015) **Implementation (of PPI Groups):** Meet in a place and time that is appropriate for young people Dewa et al. (2021) It is important to try and dissociate the sessions with the school itself Grant et al. (2020) Avoid meetings on weekends and school holidays (Children’s Council) Create an informal environment as well as a safe space for open discussions Juan et al. (2021), Miller et al. (2021), Dewa et al. (2021) Implement a rights-based framework to strengthen young people's involvement Dennehy et al. (2019) Potential honorary research status could be given to the co-researchers Dewa et al. (2021) Consider young people to co-design and co-deliver trainings to co-researchers to further reduce power restraints Dewa et al. (2021) Conduct a small on-going advisory group which is fluid and flexible Halsall et al. (2021), Dennehy et al. (2019), Dunn (2017), Grant et al. (2020), Juan et al. (2021), Kendal et al. (2017), Brady et al. (2018) *Fixed group of participants who commit to come to meetings and participate in research (Youth Council)* If a greater number of participants is needed, communicate it early Grant et al. (2020)
Phase 3	F **orms of Communication:** Involve young people in determining the best methods of communication and avoid pressuring them to participate Brady et al. (2018) Provide flexibility in communication methods such as email, telephone, and face-to-face interactions Walker et al. (2021) Maintain clear channels of communication during funding gaps to plan ahead and explore innovative ways to continue collaboration Walker et al. (2021), Miller et al. (2021) Dedicate time for team-building activities and breaks to foster a positive and interactive atmosphere Dewa et al. (2021) Ensure that young people's input is dynamic, flexible, and integrated into everyday practices and systems Walker et al. (2021), Brady et al. (2018) Use participatory enabling techniques to encourage open and honest discussions (Dennehy et al. (2019) Regularly check-in with individuals to ensure their involvement is mutually beneficial Walker et al. (2021) End meetings by seeking feedback, addressing misunderstandings, and answering questions (Children’s Council) Evaluate meetings using visual analogue scales (Children’s Council) *Communicate in simple and clear language while maintaining seriousness and relevance to the research* (Youth Council) **Data Acquisition:** Foster shared motivation to co-produce meaningful and accessible findings Walker et al. (2021) Allow young members to lead meetings, make decisions through consensus, and determine priorities Mawn et al. (2015) Generate trustworthy findings that accurately reflect young people's views without exerting undue power Kendal et al. (2017), Pullmann et al. (2013) Build rapport by establishing existing relationships within the group Grant et al. (2020) Take observational notes to analyze the influence of research development Warner et al. (2021) **Specificities Mental Health/Disorders:** Provide appropriate clinical support for young people with mental health difficulties and involve multiple co-researchers to account for potential drop-outs Dewa et al. (2021) as well as other difficulties (Parents Advisory Board) *Offer continuous support for psychological distress throughout the research process* (Youth Council) Encourage open discussions about mental health stigma and respect diverse perspectives Lincoln et al. (2015) Address challenges when forming advisory boards around mental health, such as stigma, trauma, mistrust, scheduling, confidentiality, and cultural norms Miller et al. (2021) *Ensure confidentiality regarding personal experience reports* (Youth Council) Reassure young people about the quality and safety of digital tools for mental health Grant et al., (2020), Kendal et al. (2017) Incorporate peer workers in mental health services to support the "Experts by Experience" approach Schilling et al. (2020) **Consider the remission phase as a more suitable time for participation than the acute phase of any mental disorder** (Children’s Council; Parents Advisory Board)
Phase 4	**Finalizing Study (prepare data, publications and dissemination):** Maintain a dialogue with school students to increase interest and awareness of (mental) health and epidemiological research Grant et al. (2020) *Emphasize transparency in the research process to make young people feel respected and engaged* (Youth council) Allow PPI group members to take ownership by participating in the dissemination of findings through conferences, podcasts, blog posts, and plain language summaries Walker et al. (2021) Address the ethical issue of participant anonymity versus authorship opportunities early in the process Halsall et al. (2021) **Evaluation:** Focus future research on developing a measure of youth engagement Grant et al. (2020), Pullmann et al. (2013) Conduct longer follow-up periods to study long-lasting effects on adolescents and program recipients Warner et al. (2021), Schilling et al. (2020) *Set an end point for inclusion in the research project, avoiding unnecessarily prolonged engagement* (Youth Council)

A (bold) = Parents Advisory Board; b (underlined) = Children’s Council Mental Health; c (italic) = Youth’s Council; d (small caps) = Review based;

## Results

As shown in Fig. 2, our initial searches yielded 3986 publications. 57 articles were added through the reference lists of relevant studies. After removing duplicates, 3129 publications were suitable for further evaluation. Screening titles and abstracts resulted in including 64 articles for full-text screening. Based on full-text analyses, 45 articles were excluded because no empirical research or review was provided, no CYP participated or mental health research was not conducted. Finally, 19 articles reported PPI of CYP (age range 10–26 years) in mental health research and were included for further analyses (see Table 1). The studies were from eight countries, with the majority from the UK (eight), followed by the United States of America (three) and Australia (three). One study each came from Switzerland (Pfister et al., 2021), Sweden (Warner et al., 2021), Chile (Schilling et al., 2020), Canada (Halsall et al., 2021), and the Republic of Ireland (Dennehy et al., 2019). The studies included in this systematic review were conducted between 2013 and 2022. Most studies described a multi-mixed-methods design (ten) (Dunn, 2017; Grant et al., 2020; Grové, 2020; Halsall et al., 2021; Juan et al., 2021; Kendal et al., 2017; Ospina-Pinillos et al., 2018; Pfister et al., 2021; Walker et al., 2021; Warner et al., 2021), the remaining nine took a qualitative approach (Brady et al., 2018; Dennehy et al., 2019; Dewa et al., 2021; Lincoln et al., 2015; Mawn et al., 2015; Miller et al., 2021; Pullmann et al., 2013; Schilling et al., 2020; Wright et al., 2019).

Regarding the level of participation, the majority (*n* = 14) of the included studies ranged between levels 5 (“Inclusion”) and 7 (“Partly authorized to make decisions”) (Brady et al., 2018; Dennehy et al., 2019; Dewa et al., 2021; Dunn, 2017; Grant et al., 2020; Grové, 2020; Halsall et al., 2021; Juan et al., 2021; Lincoln et al., 2015; Miller et al., 2021; Pfister et al., 2021; Pullmann et al., 2013; Schilling et al., 2020; Walker et al., 2021). Three studies stand out because they reached level 8 (“Power to make decisions”) (Kendal et al., 2017; Mawn et al., 2015; Ospina-Pinillos et al., 2018). For the extent of involvement, the majority of included studies (*n* = 12) was rated with an involvement level of 4 (co-production) (Brady et al., 2018; Dennehy et al., 2019; Dewa et al., 2021; Dunn, 2017; Grant et al., 2020; Halsall et al., 2022; Juan et al., 2021; Kendal et al., 2017; Ospina-Pinillos et al., 2018; Pullmann et al., 2013; Walker et al., 2021; Wright et al., 2019), four studies used interactive advice (level 3) (Grové, 2020; Miller et al., 2021; Schilling et al., 2020), and three of the included studies engaged in youth-led research involvement (level 5) (Lincoln et al., 2015; Mawn et al., 2015; Pfister et al., 2021). Finally, in collaboration with our Advisory Boards, we rated the participation level of our present study as a 6 (“Codetermination”) and the intensity of involvement as a 4 (“Co-production”), as CYP were actively involved in elaborating and providing the final recommendations (see Table 2 and Fig. 3).

To enable an overview of the approaches taken in PPI of CYP in mental health research, all studies were additionally evaluated considering to their model-driven approach of PPI conduct (see Table 1). The vast majority of studies (*n* = 15) provided information about their model-driven basis. The studies reviewed took nine different PPI approaches. The PPI approaches taken most often were the “Young People Advisory Group” (YPAG; *n* = 5) (Brady et al., 2018; Dewa et al., 2021; Grant et al., 2020; Juan et al., 2021; Walker et al., 2021), the “Participatory Action Research” (PAR;* n* = 3) (Pullmann et al., 2013; Schilling et al., 2020; Wright et al., 2019) and the “Community-Based Participatory Research” (CBPR; *n* = 3) (Lincoln et al., 2015; Miller et al., 2021; Pullmann et al., 2013). YPAGS are structured groups of CYP who provide input and advice on research projects. PAR is an approach involving CYP as active participants and co-researchers in the research process. CBPR is an approach specifically tailored to the collaboration between researchers and communities.

### Analysis of PPI Conduction Revealed the Following Three Types of Involvement

#### PPI as a Group Strategy

Ten out of 19 studies named advisory groups (Dennehy et al., 2019; Dewa et al., 2021; Grant et al., 2020; Juan et al., 2021; Walker et al., 2021), focus groups (Halsall et al., 2021; Juan et al., 2021; Pullmann et al., 2013; Warner et al., 2021), consultation groups (Brady et al., 2018) or youth and expert references groups (Grové, 2020) as their PPI strategy. Forming these small local groups instead of large online discussions contributed to a more open and honest discussion about study topics and mental health issues.

#### PPI at a Multilevel Approach

Six studies referred to a multilevel approach for conducting PPI (Brady et al., 2018; Dennehy et al., 2019; Dewa et al., 2021; Dunn, 2017; Grové, 2020; Juan et al., 2021). In these studies, varying levels of involvement determined by young people’s interests and their availability appeared to strengthen the collaboration. This is particularly the case when working with clinical populations.

#### PPI as Co-research

In nine out of 19 studies the participating CYP are named as co-researchers and are part of the research planning, the data collection and/or writing team or the data presenting team (Brady et al., 2018; Dewa et al., 2021; Dunn, 2017; Kendal et al., 2017; Lincoln et al., 2015; Pullmann et al., 2013; Walker et al., 2021). This type of involvement empowered young participants to decide whether they wanted to become co-researchers throughout the study process. Sharing power intensified the motivation and engagement of PPI participants.

To derive comprehensive recommendations, we additionally screened and analyzed the included papers with regard to reported barriers to the PPI of CYP. The vast majority of studies described barriers during different steps in the process. We summarized and categorized them into main barriers described either by CYP co-researchers or by professional researchers. In particular, one of the main critical aspects appears to be the extra time and personnel resources needed to organize meetings, as well as training PPI members (Dewa et al., 2021; Dunn, 2017; Mawn et al., 2015; Pfister et al., 2021). Another important barrier is reflected by the challenge to strive for continuity in participation in light of the complex and dynamic lives of CYP (Brady et al., 2018; Dennehy et al., 2019; Mawn et al., 2015; Miller et al., 2021; Pfister et al., 2021; Schilling et al., 2020). In addition, young PPI members’ lack of knowledge should be highlighted, as this affects both research methods/procedures and academic forms of communication (Lincoln et al., 2015; Pfister et al., 2021). More detailed information about the main barriers is found in the supplementary material (see Appendix A).

Finally, all recommendations extracted from the included studies were compiled and evaluated. We assigned them to the different phases of research studies: (Phase 1) Preparation and Planning including Budget; (Phase 2) Recruitment and Implementation (of groups); (Phase 3) Forms of Communication and Data Acquisition including Specificities of Mental Health/Disorders; (Phase 4) Finalizing Study and Evaluation. Our results are shown in Table 2. To enable practical and concrete implications for future studies, the recommendations were condensed and prioritized by our research team, followed by discussions and evaluations together with our three Advisory Boards. Since all three PPI groups provided their feedback on the results, the recommendations presented in Table 2 are based on a co-production process with our three Advisory Boards.

An overview of the main recommendations for CYP participation in mental health research is found in Fig. 3 based on results from the discussions with the aforementioned three PPI groups (a–c). These specific recommendations reveal the collaboration and joint development with the three PPI groups.

## Discussion

To the best of our knowledge, this is the first systematic review to have identified, analyzed, and integrated the literature on the participation of CYP in mental health research. Overall, the research involvement of CYP is growing slowly but steadily worldwide. We identified 19 studies describing initial attempts to involve CYP in mental health research. The integrated studies differed in form of PPI and in the way the participation and involvement processes were presented. However, the implications and recommendations for PPI of CYP derived from the included papers are discussed considering the PPI process and study outcomes.

### Regarding the PPI Process

According to the majority of the included studies, the main obstacle of PPI of CYP is the regular participation in the process, particularly with young people who have their own priorities that can make consistent cooperation difficult. Therefore, Brady et al. (2018), recommend a balance between maintaining contact and enabling future re-engagement, without forcing young people to participate. They suggest a fluid community of practice instead of static membership, embedding involvement in everyday practices, systems, and cultures. Young people need to be "critical friends" with the independence and resources to drive more inclusive involvement. Wright et al. (2019), emphasize respecting local contexts and working in partnership with participants in participatory action research, without forcing or rushing them. Dennehy et al. (2019) and Dewa et al. (2021) both discuss the same difficulties, but add further solutions such as participatory enabling techniques, appropriate psychological support, involving them as co-researchers, and dedicating time to creating an informal, safe environment for open discussion. Walker et al. (2021) suggest enabling flexibility in methods of contribution and maintaining regular contact to ensure mutual benefit.

Financial considerations and resource allocation appear to be other key challenges in facilitating youth participation, requiring adequate planning (e.g., using the INVOLVE calculator (Dewa et al., 2021)) and potential foundation support (Dewa et al., 2021; Pfister et al., 2021). Grant applications have already started to address this financial aspect. Ethical concerns regarding participant anonymity and inclusion are of particular importance; they need to be addressed by providing different opportunities for PPI members to contribute to the dissemination of research findings through various mediums. The lack of knowledge among young PPI members is another significant barrier, highlighting the need for improved communication between researchers and youth (Lincoln et al., 2015; Pfister et al., 2021). Nonetheless, this challenge presents an opportunity for mutual benefits, including tailored mental health interventions, inclusivity, and in reducing stigma and promoting resilience.

In four studies, young people reported barriers to and suggestions for facilitating PPI of CYP. They suggest strategies to overcome barriers and enhance PPI, such as involving young individuals with lived experiences, incorporating their perspectives, and employing flexible communication methods (Brady et al., 2018). The young people’s recommendations also include allowing personal clothing choices (Dennehy et al., 2019), providing more information sessions, exploring additional topics, shorter breaks in participation, skills-based training, and utilizing small groups for PPI of CYP (Dewa et al., 2021; Dunn, 2017).

Besides these barriers, several main implications can be drawn from the included studies: First, almost every paper addresses the necessity to create a safe, comfortable and youth-friendly environment for participants. This is especially important when discussing their topics and priorities (Juan et al., 2021), accessing digital tools for emotional health (Kendal et al., 2017) or when it comes to mental health topics in general. Second, the conduction of participation ranged from small local groups to online data collections. More detailed information about forming participation groups and group sizes was not provided. However, the most important goal remains to establish trust and transparency in the discussions with youth participants (Lincoln et al., 2015; Miller et al., 2021), which is best achieved in small local groups or individual consultations rather than large online-discussion settings. Third, participation should be quite flexible and fluid, and different forms of contribution should be considered (Brady et al., 2018; Grant et al., 2020). Finally, young participants need special guidance at all steps in the process: information about the purpose and the conduction of the study as well as data analyses and evaluation should be prepared in simple and age-specific language and presented transparently (Walker et al., 2021). Unfortunately, our analysis of the included studies also revealed some significant limitations that future studies will have to address. In particular, we noticed that there are some countries in which PPI of CYP, and participation in general, is developing and advancing more rapidly. In particular, the UK, USA and Australia have a clear lead over Europe in this respect. These findings are in line with previous research about PPI of adult participants (Biddle et al., 2021).

### Outcome of Studies

A main distinct limitation of these studies, however, is the lack of participation outcome measurements. The included studies report no quantitative data about their experiences in participation, which reflects a desperate need for operationalizing a measure of youth engagement that is actionable, measurable, and associated with outcomes (Pullmann et al., 2013). These studies’ heterogeneity made a comparison difficult per se. The stage model by Wright et al. is a helpful instrument for determining together with CYP which form of participation should be implemented. Subsequently, an assessment can be made collaboratively to determine whether this stage has been reached and whether it has been helpful for participation at this point of the research process. In addition, hardly any of the included studies described when and to what extent the young people’s decisions changed the opinion of the researchers. Here, the study by Walker and colleagues (Walker et al., 2021) stands out in a positive light, as it offers a clear and concise overview of the influence of CYP on each steps in the study process. We therefore proceeded similarly and marked the additional recommendations from our advisory groups (see Table 1). Our intention, here, is to contribute to future studies by promoting the adoption of a similar approach, whereby investigators acknowledge and highlight the valuable input that PPI members provide. Evaluating the impact of PPI of CYP is crucial for understanding its effectiveness and potential benefits. Therefore, suitable quantitative measures need to be employed to evaluate the extent of PPI of CYP, such as tracking the number of involvement activities undertaken, assessing the feedback and input of those involved, and the level of satisfaction reported by researchers and participants. Especially concerning tokenisms (where PPI participants only serve as representatives lacking real power to make decisions), the results of discussions need to be transparent. Elaborating such measurements in co-production with CYP would increase their usability and comprehensibility, which would not benefit both CYP participants and researchers. Combining these quantitative approaches with the qualitative information gathered through the PPI process itself enables a comprehensive evaluation of PPI of CYP, providing valuable insights into its value and potential for improving future mental health research practices. Furthermore, several researchers indicate that adolescent participation in projects and programs should entail a longer-term evaluation to investigate possible long-lasting effects on both the CYP and on the eventual recipients of the programs and treatments (Grant et al., 2020; Schilling et al., 2020; Warner et al., 2021).

Finally, it is noteworthy that hardly any studies are carried out involving the participation of younger children. Although mental health research studies are being conducted with very young children worldwide, the youngest age of the included studies here was ten years (Walker et al., 2021). The vast majority of studies therefore included adolescents. However, younger children, i.e. pre-schoolers and primary-schoolers, should not be neglected in PPI. Mental health problems often emerge in childhood and adolescence, and early intervention is crucial to prevent long-term negative outcomes. Therefore, giving young children a voice will lead to a better understanding of how mental health issues develop, and whether measures and treatment are developmentally appropriate and effective for children of different ages and backgrounds. Additionally, involving children in mental health research empowers them and can help reduce stigma around mental health as early as possible. Experiences with our children’s advisory group have been consistently positive. They willingly evaluated the results of our review and provided additional helpful input. Therefore, we encourage mental health researcher to also address young children in the future.

To sum up, the results of our systematic review provide an overview of existing participatory CYP research. A systematic inclusion of CYP above and beyond mere questioning addresses the necessity of bringing research to the people and most likely facilitates acceptance, implementation and the dissemination of research in the populations of interest. Despite the fact that the vast majority of the included papers reported hurdles in implementing PPI of CYP, the additional benefit outweighs the extra work in all studies. CYP are important partners in mental health research: They provide their unique feedback, opinions and views on considerations that adult researchers will never be able to fully elaborate on their own. In addition, CYP can actively participate in research tasks, i.e. by helping to recruit peer participants, in the choice of data collection, or in helping to disseminate materials (Mawn et al., 2015). PPI of CYP can thus benefit both mental health research and the underage participants together with the professional researchers involved. The extent of participation and involvement was rated quite high overall. Even if the highest level of participation (self-management) was been achieved by any of the included studies, it is also unclear to what extent that participation level can be achieved in mental health research at all, particularly in CYP research. With regard to the level of involvement, the first attempts to empower young people as co-researchers proved to be feasible and beneficial. Therefore, the extent and intensity of PPI of CYP in the included studies are exemplary for the first attempts to include CYP in mental health research.

## Limitations

The following limitations must be considered. Foremost, a structured analysis of empirical data for comparing different PPI strategies was not possible due to the heterogeneity of the available data. The decision to cluster studies based on temporal phases was subsequently made by the study team and not collaborated on by the Advisory Boards at this point. Furthermore, the extracted studies were not analyzed with respect to different mental disorders, as we only evaluated their approaches. Future studies could take this aspect into account. Finally, we did not apply co-production techniques in the phase of decision-making of search terms.

## Strengths

A strength of our study lies in its inclusive and comprehensive approach. By actively engaging the perspectives of all stakeholders, including younger children, adolescents and parents, our study ensures a holistic understanding of the challenges and opportunities associated with PPI in this context. This participatory methodology promotes a more nuanced and relevant set of recommendations. Our study's commitment to involving diverse voices reflects a commendable effort to bridge the gap between even complex research, such as systematic reviews, and the lived experiences of the younger populations and their families.

## Conclusion

The involvement of CYP in mental health research is crucial for several reasons. Firstly, it acknowledges that young individuals are experts in their own experiences and perspectives. By actively involving them in research, their voices, needs, and preferences can be better understood and incorporated in developing effective interventions and treatments. Secondly, engaging CYP in mental health research empowers them to improve their autonomy and self-advocacy skills. It gives them to have a say in matters that directly impact their mental well-being, fostering a sense of ownership and control over their own lives. Involving CYP in research also contributes to the ethical conduct of studies by ensuring that their rights, safety, and welfare are protected. Including CYP in mental health research encourages early intervention and prevention strategies, which can lead to improved outcomes and long-term mental health resilience. Overall, their involvement is vital to enabling comprehensive and age-appropriate approaches to mental health care. Although PPI of CYP needs to be optimized and accompanied by the collection of quantitative data, only by including them as equal participants can future research reflect the tenets of holistic science. Recommendations to improve PPI of CYP should therefore be taken into account in future research designs in mental health research to support translation-back translation methodology. Ensuring the empirical accompaniment of PPI is crucial in order to effectively measure its benefits. To achieve this, we need to develop suitable measurement instruments.

### Supplementary Information

Below is the link to the electronic supplementary material.

Supplementary file1 (PDF 93 kb)

Supplementary file2 (DOCX 27 kb)

Supplementary file3 (DOCX 15 kb)
