# Supplementary figures and images for: Systematic Review: Patient and Public Involvement of Children and Young People in Mental Health Research
Source: Clin Child Fam Psychol Rev. 2024 Feb 25;27(1):257–74. doi: 10.1007/s10567-024-00470-x (PMC10920437; doi:10.1007/s10567-024-00470-x)

## Reported Barriers to PPI of Children and Young People in Mental Health Research

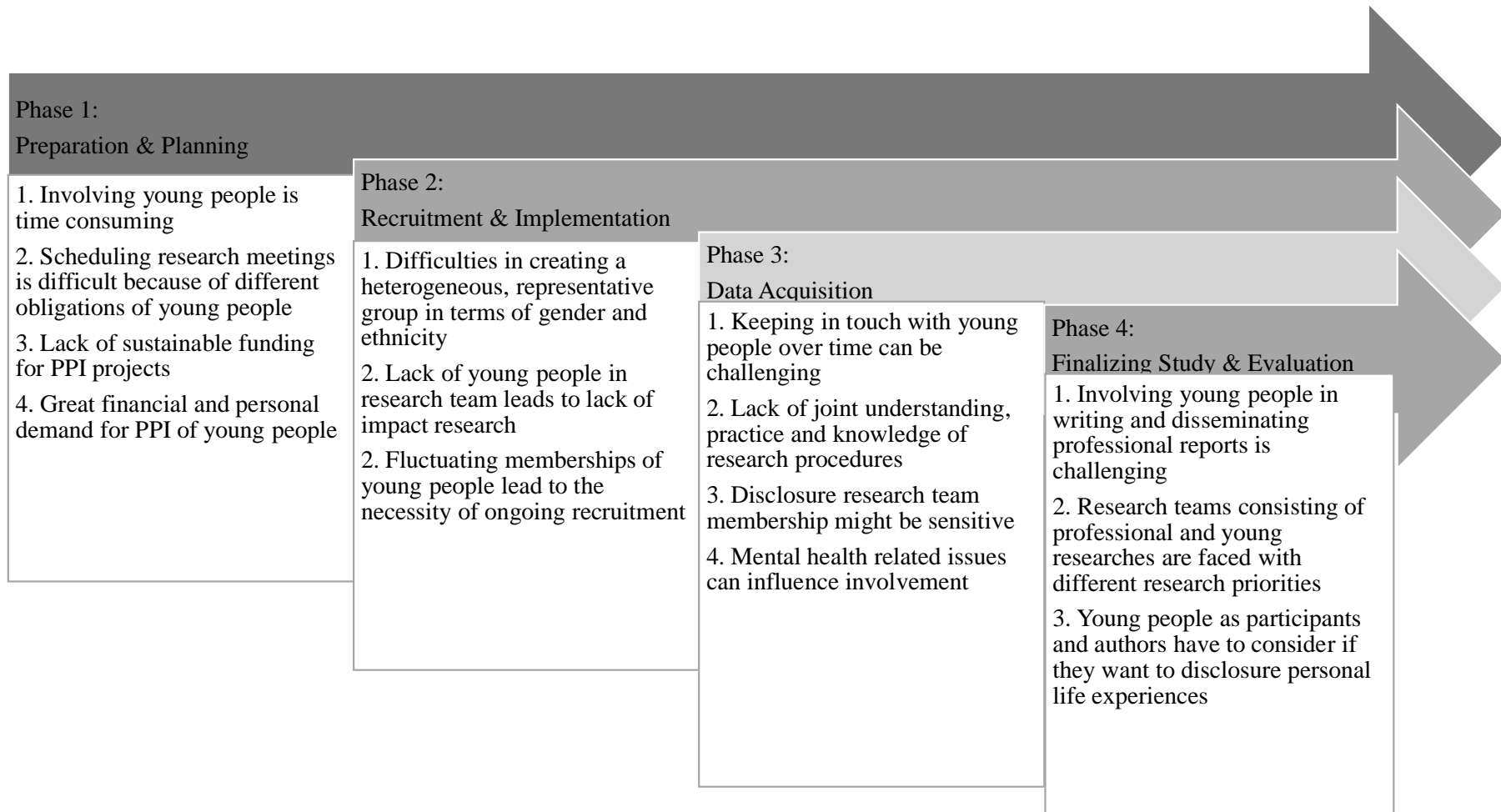

Supplement: Supplementary file 1 — Supplementary file1 (PDF 93 kb) [file 10567_2024_470_MOESM1_ESM.pdf]
