# Supplementary material for: Systematic Review: Patient and Public Involvement of Children and Young People in Mental Health Research
Source: Clin Child Fam Psychol Rev. 2024 Feb 25;27(1):257–74. doi: 10.1007/s10567-024-00470-x (PMC10920437; doi:10.1007/s10567-024-00470-x)
Supplement: Supplementary file 3 — Supplementary file3 (DOCX 15 kb) [file 10567_2024_470_MOESM3_ESM.docx]

**PubMed**

1. “((Patient participation[Mesh]) OR (community-based participation research[Mesh])) OR ((patient involvement[Title/Abstract]) OR (public involvement[Title/Abstract]) OR (patient engagement[Title/Abstract]) OR (PPI[Title/Abstract]) OR (participatory research[Title/Abstract]))”

2. “(child*[Title/Abstract]) OR (youth[Title/Abstract])”

3. “1 AND 2“

4. “(Youth involvement[Title/Abstract]) OR (child* involvement[Title/Abstract]) OR (child* engagement[Title/Abstract]) OR (youth engagement[Title/Abstract]) OR (young person* advisory group [Title/Abstract]) OR (YPAG[Title/Abstract])”

5. ”3 OR 4”

6. “(Mental health research[Title/Abstract]) OR (clinical research[Title/Abstract])”

7. “5 AND 6”

**PsycInfo**

Selected Databases: APA PsycInfo, APA PsycArticles, APA PsycBooks, APA PsycExtra (all selected)

1**. First Page**:” patient involvement *OR* **First Page**: public involvement *OR* **First Page**: youth involvement *OR* **First Page**: child* involvement *OR* **First Page**: patient engagement *OR* **First Page**: child* engagement *OR* **First Page**: youth engagement *OR* **First Page**: PPI *OR* **First Page**: young person* advisory group *OR* **First Page**: YPAG *AND* **Year**: 2000 *To* 2022”

2. **First Page**:” participatory research *OR* **First Page**: Child* led research *OR* **First Page**: youth led research *AND* **Year**: 2000 *To* 2022”

3. **MeSH**:” patient participation *OR* **MeSH**: community-based participatory research *OR* **MeSH**: research personal”

4. **MeSH**:“ mental health“

5. „3 AND 4”

**PSYNDEX**

1. ”(((Patient participation[Mesh]) OR (community-based participation research[Mesh])) AND (Mental Health[Mesh])) OR (((patient involvement) OR (public involvement) OR (youth involvement) OR (child* involvement) OR (patient engagement) OR (child* engagement) OR (youth engagement) OR (PPI) OR (Young person* advisory group) OR (YPAG)) AND ((mental health research) OR (clinical research))) OR ((Participatory research) OR (Child led research) OR (Youth led research))”

**Web of Science**

1. “Patient participation or community-based participation research or patient involvement or public involvement or patient engagement or PPI or participatory research”

2. „Child* or youth”

3. „1 AND 2“

4. “Youth involvement or child* involvement or child* engagement or youth engagement or young person* advisory group or YPAG”

5. „3 OR 4“

6. “Mental health research or clinical research”

7. „5 AND 6“
